# The Complexity of SIRT2 in Chronic Liver Disease: Liver SIRT2 Promotes Hepatocellular Carcinoma Development

**DOI:** 10.1016/j.jcmgh.2025.101512

**Published:** 2025-04-22

**Authors:** Xinjian Li, Shaolei Lu, Chiung-Kuei Huang

**Affiliations:** Department of Pathology and Laboratory Medicine, Tulane University School of Medicine, New Orleans, Louisiana; Department of Pathology and Laboratory Medicine, Warren Alpert Medical School of Brown University, Rhode Island Hospital, Providence, Rhode Island; Department of Pathology and Laboratory Medicine, Tulane University School of Medicine, New Orleans, Louisiana

Hepatocellular carcinoma (HCC) is one of the leading causes of cancer-related mortality worldwide. Its development is associated with several risk factors, including alcohol-associated liver disease (ALD), hepatitis virus infections, metabolic dysfunction-associated steatotic liver disease (MASLD), and exposure to environmental toxins. Early-stage HCC diagnosis offers access to curative-intent treatments such as ablation, liver transplantation, and surgical resection, which are associated with a 5-year survival rate exceeding 70%.^1^ However, the absence of reliable early diagnostic markers often results in late-stage diagnoses, limiting the applicability of these curative options. For advanced HCC, sorafenib has been the first-line systemic therapy for over a decade.^2^ Recent advancements have shown that combining a programmed death-ligand 1 (PD-L1) inhibitor (atezolizumab) with a vascular endothelial growth factor (VEGF) monoclonal antibody (bevacizumab) improves progression-free and overall survival in treatment-naïve patients with HCC compared with sorafenib.^3^ Despite these improvements, the 28-month overall survival rate remains at 36%,^4^ raising questions about the long-term efficacy of this combination therapy in achieving a 5-year survival benefit.^4^ Therefore, it is essential to develop novel therapeutic targets for patients with advanced HCC by elucidating the molecular mechanisms driving HCC progression.

Sirtuins (SIRTs) are a conserved family of NAD^+^-dependent protein deacetylases that regulate physiologic processes and disease progression through epigenetic and non-epigenetic mechanisms. An epigenetic regulator can function as an oncogene^5^ or tumor suppressor,^6^ depending on cell and tissue types. It has been previously demonstrated that knockout (KO) of whole-body SIRT2 in mice would cause tumorigenesis in the liver, mammary, lung, and gastrointestinal tract tissues.^7^ However, it was reported that the expression of SIRT2 is elevated in human HCC samples, and high SIRT2 expression is correlated with poor prognosis in patients with HCC. Besides, inhibiting SIRT2 results in suppressing HCC progression.^8^ Further, SIRT2 KO could protect mice from carbon tetrachloride and thioacetamide-induced liver fibrosis/cirrhosis, which could lead to HCC or liver failure.^9^ Due to these controversial observations, it is necessary to further understand tissue-specific SIRT2’s role in HCC development. In this issue of *Cellular and Molecular Gastroenterology and Hepatology*, Wang, Keating, and Xu et al investigated the liver-specific SIRT2 role in HCC initiation and progression.^10^ Although liver-specific SIRT2 KO had minimal impact on normal liver development, it significantly suppressed HCC progression in 2 models induced by c-Met/β-Catenin and myrAKT/NRasG12V overexpression, respectively. Mechanistically, it was suggested that the SIRT2-mediated liver tumorigenesis is likely through the C/EBPβ/GADD45γ signaling cascade. These findings were sophistically demonstrated by the rescuing experiments that knockdown of C/EBPβ or GADD45γ reverses the impact of SIRT2 inhibition on HCC progression and that overexpression of C/EBPβ or GADD45γ suppresses HCC development related to SIRT2 upregulation. Besides, the results of SIRT2 inhibitor experiments also revealed that targeting SIRT2 in human HCC cells could reduce cell proliferation, thus supporting the potential of targeting SIRT2 in treating patients with HCC. Similar findings that SIRT2 is negatively associated with C/EBPβ/GADD45γ were found in a specific subset of patients with HCC, further strengthening the importance of understanding SIRT2, C/EBPβ, and GADD45γ levels in human patients with HCC before applying the SIRT2 inhibitor in human clinical trials. Collectively, this study establishes SIRT2 as a promoter of HCC progression, particularly in patients with dysregulated AKT and β-catenin signaling pathways.^11^

SIRT2 is a potentially novel target in patients with advanced HCC. Nevertheless, several future directions may warrant further investigation. It is important to investigate whether SIRT2 inhibitors are effective in preclinical HCC models. It remains unclear how SIRT2 is upregulated in patients with advanced HCC. Clarifying how SIRT2 is upregulated in patients with advanced HCC may help identify patient subgroups most likely to benefit from SIRT2-targeted therapies. Besides, understanding the molecular mechanisms by which SIRT modulates C/EBPβ and GADD45γ may help identify novel targeting strategies. Recent studies have suggested the involvement of SIRT2 in resistance to immunotherapy and angiogenesis function in cancer progression.^12^^,^^13^ Given that the treatment of atezolizumab plus bevacizumab has been suggested to be the first-line of standard care for advanced HCC, it will be exciting to figure out the impacts of SIRT2 on immunotherapy, anti-VEGF monoclonal antibody treatment, and the combination of PD-L1 inhibitor and anti-VEGF monoclonal antibody in treating advanced HCC. Furthermore, SIRT2 plays protective roles in inflammation,^14^ MASLD,^15^ and ALD,^16^ contradicting its tumor promoter roles in patients with HCC with dysregulated AKT and β-catenin signaling pathways. To broaden its potential use in most patients with HCC linked to these chronic liver diseases, it will also be interesting to investigate how SIRT2 impacts the development of HCC associated with ALD, hepatitis, and MASLD.
